# Gender and Leadership Trajectories in Academic Dentistry

**DOI:** 10.1111/eje.70109

**Published:** 2026-02-12

**Authors:** Nikkita Madhu, Divya Kohli, Linda Sangalli

**Affiliations:** ^1^ Department of Prosthodontics, College of Dentistry University of Iowa Iowa City Iowa USA; ^2^ Department of Oral and Maxillofacial Surgery College of Dental Medicine, Nova Southeastern University Davie Florida USA; ^3^ College of Dental Medicine – Illinois, Midwestern University Downers Grove Illinois USA

**Keywords:** academic dentistry, dental faculty, dentistry, gender disparity, leadership

## Abstract

**Objectives:**

Despite increased number of women entering dental academia in the last decade, gender disparities persist in leadership roles. This study examined gender trends within administrative leadership across U.S. dental schools.

**Materials and Methods:**

Data on gender, age, employment status, academic rank and administrative title were extracted from the ADEA Survey of Dental School Faculty. Prevalence ratios (PRs) and 95% confidence intervals (CIs) were calculated to assess gender representation in leadership over time, stratified by rank and age and adjusted for employment status (aPR).

**Results:**

On average, women held fewer leadership roles than men (17.5% vs. 19.8%, *p* < 0.001), even after adjusting for employment status. Women were underrepresented in senior administrative positions such as department chairs (PRs = 0.57–0.81), division directors (PRs = 0.40–0.72) and deans (PRs = 0.46–0.62). Younger women were more likely to hold leadership roles than those aged ≥ 60.

**Conclusion:**

While overrepresented as allied program directors and other nonspecific directorships, women continue to be underrepresented in more senior leadership roles, underscoring the need for systemic reform. Women continue to hold fewer senior leadership roles than men, emphasising the need for systemic change in policies and institutional culture to facilitate gender equity in academic dentistry.

## 1 Introduction

There has been a steady rise in the percentage of women enrolled in dental schools and advanced dental education programs over the last decade [1]. Women are more likely to choose academic careers compared to their male counterparts [2], which translates to more women in entry‐level, junior ranks in dental education. This trend was evident in prior work that noted that despite this, women still advanced to senior positions slower than men [3].

Both within and outside the dentistry, women experience inequities in professional advancement [4, 5, 6]. Similar to the ‘leaky pipeline’ [7], the concept of vertical gender segregation comes up in literature around women advancing their careers in healthcare [8, 9]. At the base of the hierarchal occupational ‘ladder’, there may be close to equal distributions of men and women. However, as we vertically go up the ‘ladder’, the distribution in leadership positions quickly skews in favour of men, as does remuneration [10, 11].

This disparity is a product of a vicious cycle: the lack of female representation in the upper echelons of academic healthcare is perpetuated by the consequent lack of role models and mentorship opportunities for young female faculty interested in leadership positions and vice versa [12]. Advancing academic rank is also historically based on scholarly activity. However, there remains documented inequity in research productivity. Women have lower publication rates than men [13], are less frequently first authors [14] and are less likely to receive federal grants and awards [15]. Several factors contribute to this lower productivity rate, including career gaps, childbearing and family care, among others [16]. Since most of these factors play a key role in consideration for promotion to higher ranks and senior roles, it could ultimately negatively impact the number of women in administrative leadership roles.

These disparities in pay, career advancement and work distribution ultimately force women in academic healthcare to either reduce their hours or leave academic healthcare altogether [17]. Women appear more likely to reduce employment to part‐time than men due to difficulties establishing work‐life balance in the face of existing challenges to career growth, that were exacerbated by the COVID‐19 pandemic [18]. This consequentially, limits how likely these women would be considered for full‐time leadership roles.

Recent initiatives to promote the representation of minority groups and faculty from diverse backgrounds, such as the American Dental Education Association (ADEA) Leadership Institute [19], ADEA/WKKF Minority Faculty Development program [20], ADEA diversity toolkit [21] and American Dental Association (ADA) Institute for Diversity in Leadership [22], among others, represent promising strategies to drive systematic change within dental academia.

The aim of this study was to examine gender trends among administrative leadership roles in dental education after adjusting for employment status between 2011 and 2023, as it relates to age and academic rank. We hypothesized that more women would hold senior leadership in dental education over time, with upward trends concentrated in specific age groups rather than occurring uniformly across all ages. Our results could inform the development of initiatives to increase diversity in academic leadership.

## 2 Materials and Methods

### 2.1 Study Design

This study was considered exempt from the Institution Review Board (IRB) of the University of Iowa (IRB 202501444). The study adhered to the ADEA's data‐use agreement and ethical guidelines. Consistent with this agreement, the current data were used solely for research purposes to evaluate trends in gender representation.

### 2.2 Data Collection

This study is a secondary analysis of data previously collected through the ADEA Survey of Dental School Faculty, ranging from 2011–12 to 2022–23 academic years. Data collected included 66 U.S. CODA‐accredited dental schools that participated in the ADEA survey in the study period. Available data included the academic years from 2011–2012 to 2022–2023, with the exception of 2019–2020 and 2020–2021 academic years, which were missing due to the survey's redesign during these 2 years.

### 2.3 Study Variables

The data consisted of de‐identified, aggregated information obtained from ADEA. The variables evaluated were:

- Gender, classified as man, woman, transgender man, transgender woman, genderqueer/gender non‐conforming, different gender identity, gender unknown/cannot report.
- Age, initially categorised into eight groups: 20–29, 30–39, 40–49, 50–59, 60–69, 70–79, 80–89 and 90 years and older. These were consolidated into three broader categories of 20–39, 40–59 and 60 years and above.
- Employment status, listed as full‐time (≥ 0.5 full‐time equivalent, FTE) or part‐time (< 0.5 FTE).
- Academic rank, listed as Professor, Associate Professor, Assistant Professor, Instructor, Lecturer, Teaching/Research Associate or Assistant, Professor, Professor Emeritus, and Other. These categories were also analysed as belonging to junior ranks (i.e., Instructor, Lecturer and Assistant Professor) and senior ranks (i.e., Associate Professor, Professor and Professor Emeritus). The rank of Teaching/Research Associate or Assistant was omitted from the analysis into junior versus senior academic rank as the category combined both junior and senior ranks.
- Administrative title, expressed as a dichotomous variable (yes/no) and administrative position. An administrative title was defined as > 80% of time assigned to non‐instructional and non‐research activities. Those respondents who indicated holding an administrative position were further provided the option to select among President, Vice President, Dean Emeritus, Dean, Acting Interim Dean, Associate Dean, Assistant Dean, Program Director, Allied Dental Program Director, Clinic Director, Division Director, Other Director, Department Chair, Other Administrative Position or Professor.

### 2.4 Statistical Analysis

Descriptive statistics were used to summarise the proportion of women and men with leadership positions and across each role during the study period (2011–12 to 2022–23). Differences in proportion between women and men were calculated through chi‐square test using 2 × 2 contingency tables. To quantify relative representation of gender in leadership positions over time, prevalence ratios (PRs) and 95% confidence intervals (CIs) were calculated. PRs were computed by dividing the proportion of women (or men) in a specific leadership role by their proportion among all faculty members holding leadership roles in the corresponding year. A PR of 1 indicates equal representation; PRs > 1 indicate overrepresentation, and PRs < 1 indicate underrepresentation of women relative to their male counterparts. Next, we computed PRs stratified by academic rank to examine gender representation within leadership positions across career stages. For each academic rank and year, PRs were calculated by dividing the proportion of women in leadership roles within that academic rank by their proportion in leadership roles across all other ranks, and then comparing this to the corresponding proportion among men. This approach allowed us to assess women's representation in each rank relative to men, controlling for the distribution of leadership roles across all ranks within each year.

To account for differences in full‐time versus part‐time employment, we conducted a direct standardisation of leadership role prevalence by adjusting the male faculty distribution to match the full‐time and part‐time composition of the female faculty group. Specifically, we applied the proportion of full‐time and part‐time employment observed among women across the study period to the male group's leadership prevalence, thereby estimating an adjusted prevalence ratio (aPR) for women compared to men, independent of employment status. This method allowed us to assess whether the observed gender gap in leadership role could be attributed to differences in full‐time versus part‐time employment.

Finally, gender distribution in leadership roles was analysed across age groups. For each group, PRs and 95% CIs were calculated by comparing the proportion of women versus men holding leadership roles, with similar interpretation of PRs as described above.

PRs and 95% CIs were computed with OpenEpi v.3, TwobyTwo ‘Risk Based’ estimates.

## 3 Results

The ADEA U.S. Dental School Faculty Census included a total of 101 359 entries, averaging 10 136 respondents per year, with annual totals ranging from 10 103 in 2011–12 to 9011 in 2022–23. Respondents self‐identified their gender as men, women, or unknown/not reported. Entries with unspecified gender (‘Unknown/not reported’, *n* = 1591) or missing information regarding administrative roles (‘Unknown’, *n* = 90) were excluded, resulting in a final sample of 99 678 records (63.9% men and 36.1% women).

### 3.1 Gender Distribution According to Leadership Roles

Over the study period, an average of 17.5% of women (89.5% of them working full‐time) held leadership roles, in comparison with 19.8% of men (84.0% of them working full‐time) (*X* ^2^ (1, *N* = 99 678) = 83.2, *p* < 0.001). There was a statistically significant change in gender distribution in leadership roles over the study period (*X* ^2^ (1, *N* = 3684) = 102.0, *p* < 0.0001). Specifically, in the 2011–12 academic year, 17.3% of women held leadership positions compared to 21.2% of men (PR = 0.84, 95% CI 0.78, 0.91). This proportion decreased to 16.2% in 2015–16 but subsequently increased, eventually surpassing that of men in 2021–22, when 20.6% of women held leadership roles compared to 19.2% of men (PR = 1.05, 95% CI 0.99, 1.12, Table 1). After adjusting for full‐time versus part‐time employment, the aPR was 0.88, indicating that women remained underrepresented in leadership roles even after controlling for employment status. Figure 1 illustrates these trends over time.

**TABLE 1:** Gender distribution of leadership roles over time.

Leadership role	Gender	2011–12	2012–13	2013–14	2014–15	2015–16	2016–17	2017–18	2018–19	2021–22	2022–23	% change^a^
Allied Dental Program Director	Women	16 (0.8%)	17 (0.9%)	18 (0.9%)	18 (0.9%)	18 (0.9%)	20 (1.1%)	23 (1.2%)	22 (1.2%)	0 (0.0%)	0 (0.0%)	−100.0%
	Men	6 (0.3%)	4 (0.2%)	2 (0.1%)	1 (0.1%)	1 (0.1%)	1 (0.1%)	1 (0.1%)	1 (0.1%)	0 (0.0%)	0 (0.0%)	−100.0%
	PRs (95% CI)	2.71 (2.08, 3.53)	2.72 (2.19, 3.39)	3.04 (2.59, 3.57)	3.27 (2.88, 3.71)	3.09 (2.72, 3.50)	3.05 (2.71, 3.43)	2.94 (2.65, 3.27)	2.84 (2.55, 3.17)	n/a	n/a	
Program Director, Other Program Director	Women	76 (3.6%)	87 (4.4%)	74 (3.5%)	72 (3.7%)	73 (3.7%)	68 (3.7%)	72 (3.7%)	70 (3.8%)	178 (11.4%)	170 (9.2%)	155.6%
	Men	144 (6.9%)	172 (8.8%)	148 (7.0%)	139 (7.1%)	129 (6.6%)	114 (6.2%)	132 (6.8%)	122 (6.7%)	216 (13.9%)	217 (11.7%)	69.6%
	PRs (95% CI)	1.30 (1.07, 1.59)	1.13 (0.94, 1.36)	1.12 (0.92, 1.36)	1.17 (0.96, 1.44)	1.18 (0.97, 1.43)	1.19 (0.97, 1.46)	1.07 (0.87, 1.30)	1.07 (0.88, 1.30)	1.03 (0.91, 1.17)	1.11 (0.97, 1.26)	
Clinic Director	Women	68 (3.3%)	75 (3.8%)	89 (4.2%)	89 (4.5%)	87 (4.5%)	94 (5.1%)	106 (5.5%)	111 (6.1%)	0 (0.0%)	0 (0.0%)	−100.0%
	Men	159 (7.6%)	191 (9.8%)	206 (9.8%)	195 (9.9%)	198 (10.2%)	196 (10.6%)	206 (10.6%)	205 (11.2%)	0 (0.0%)	170 (9.2%)	−21.5%
	PRs (95% CI)	1.11 (0.90, 1.37)	0.92 (0.75, 1.13)	1.00 (0.83, 1.20)	1.07 (0.86, 1.29)	0.97 (0.80, 1.17)	1.02 (0.85, 1.262)	1.02 (0.86, 1.21)	1.02 (0.87, 1.21)	n/a	n/a	
Division Director	Women	26 (1.2%)	30 (1.5%)	29 (1.4%)	28 (1.4%)	31 (1.6%)	29 (1.6%)	30 (1.5%)	31 (1.7%)	0 (0.0%)	0 (0.0%)	−100.0%
	Men	195 (9.3%)	102 (5.2%)	103 (4.9%)	101 (5.1%)	91 (4.7%)	84 (4.5%)	92 (4.7%)	100 (5.5%)	0 (0.0%)	0 (0.0%)	−100.0%
	PRs (95% CI)	0.40 (0.28, 0.58)	0.74 (0.53, 1.02)	0.71 (0.51, 0.99)	0.72 (0.51, 1.01)	0.80 (0.58, 1.09)	0.79 (0.57, 1.09)	0.72 (0.53, 0.99)	0.67 (0.49, 0.92)	n/a	n/a	
Other Director	Women	108 (5.2%)	118 (6.0%)	120 (5.7%)	124 (6.3%)	131 (6.7%)	117 (6.3%)	143 (7.4%)	133 (7.3%)	192 (12.3%)	233 (12.6%)	142.3%
	Men	177 (8.5%)	94 (4.8%)	199 (9.4%)	212 (10.8%)	206 (10.6%)	193 (10.4%)	207 (10.7%)	185 (10.1%)	212 (13.6%)	250 (13.5%)	58.8%
	PRs (95% CI)	1.48 (1.45, 1.75)	2.05 (1.77, 2.36)	1.30 (1.11, 1.53)	1.31 (1.12, 1.54)	1.31 (1.12, 1.52)	1.23 (1.04, 1.44)	1.29 (1.11, 1.49)	1.27 (1.10, 1.48)	1.10 (0.98, 1.25)	1.27 (1.14, 1.43)	
Department Chair	Women	98 (4.7%)	54 (2.8%)	93 (4.4%)	60 (3.0%)	67 (3.4%)	65 (3.5%)	72 (3.7%)	75 (4.1%)	101 (6.5%)	102 (5.5%)	17.0%
	Men	396 (19.0%)	241 (12.3%)	275 (13.0%)	240 (12.2%)	230 (11.8%)	212 (11.5%)	224 (11.6%)	204 (11.2%)	177 (11.4%)	173 (9.3%)	−51.1%
	PRs (95% CI)	0.67 (0.55, 0.81)	0.57 (0.44, 0.73)	0.81 (0.67, 0.98)	0.64 (0.50, 0.81)	0.69 (0.55, 0.86)	0.70 (0.56, 0.88)	0.70 (0.56, 0.86)	0.75 (0.61, 0.92)	0.79 (0.67, 0.93)	0.90 (0.76, 1.06)	
Assistant Dean	Women	24 (1.1%)	27 (1.4)	27 (1.3%)	31 (1.6%)	36 (1.8%)	36 (1.9%)	40 (2.1%)	46 (2.5%)	48 (3.1%)	55 (3.0%)	−172.7%
	Men	46 (2.2%)	57 (2.9%)	61 (2.9%)	59 (3.0%)	60 (3.1%)	59 (3.2%)	53 (2.7%)	45 (2.5%)	33 (2.1%)	47 (2.5%)	−13.6%
	PRs (95% CI)	1.27 (0.91, 1.77)	1.07 (0.77, 1.46)	1.02 (0.74, 1.40)	1.17 (0.87, 1.57)	1.21 (0.93, 1.58)	1.20 (0.92, 1.56)	1.31 (1.03, 1.67)	1.50 (1.21, 1.86)	1.37 (1.13, 1.65)	1.36 (1.12, 1.64)	
Associate Dean	Women	68 (3.3%)	79 (4.0%)	81 (3.8%)	80 (4.1%)	87 (4.5%)	83 (4.5%)	92 (4.7%)	84 (4.6%)	108 (6.9%)	119 (6.4%)	93.9%
	Men	129 (6.2%)	167 (8.5%)	158 (7.5%)	154 (7.8%)	155 (8.0%)	144 (7.8%)	155 (8.0%)	144 (7.9%)	122 (7.8%)	132 (7.1%)	14.5%
	PRs (95% CI)	1.30 (1.06, 1.60)	1.07 (0.88, 1.30)	1.14 (0.94, 1.38)	1.18 (0.97, 1.43)	1.17 (0.98, 1.41)	1.17 (0.97, 1.41)	1.14 (0.95, 1.35)	1.08 (0.90, 1.30)	1.07 (0.92, 1.25)	1.20 (1.04, 1.39)	
Dean^b^	Women	12 (0.6%)	9 (0.5%)	13 (0.6%)	13 (0.7%)	13 (0.7%)	11 (0.6%)	11 (0.6%)	12 (0.7%)	17 (1.1%)	17 (0.9%)	50.0%
	Men	46 (2.2%)	55 (2.8%)	51 (2.4%)	48 (2.4%)	48 (2.5%)	45 (2.4%)	48 (2.5%)	43 (2.4%)	42 (2.7%)	50 (2.7%)	22.7%
	PRs (95% CI)	0.75 (0.45, 1.25)	0.46 (0.25, 0.84)	0.67 (0.41, 1.09)	0.71 (0.44, 1.16)	0.67 (0.41, 1.10)	0.61 (0.36, 1.04)	0.55 (0.32, 0.94)	2.20 (1.65, 2.94)	0.64 (0.43, 0.96)	0.62 (0.41, 0.93)	
Vice President	Women	0 (0.0%)	0 (0.0%)	0 (0.0%)	0 (0.0%)	0 (0.0%)	0 (0.0%)	3 (0.2%)	0 (0.0%)	0 (0.0%)	1 (0.1%)	0.0%
	Men	0 (0.0%)	1 (0.1%)	1 (0.1%)	1 (0.1%)	1 (0.1%)	1 (0.1%)	2 (0.1%)	0 (0.0%)	0 (0.0%)	0 (0.0%)	0.0%
	PRs (95% CI)	n/a	n/a	n/a	n/a	n/a	n/a	1.80 (0.88, 3.70)	n/a	n/a	n/a	
Other Administrative Position	Women	57 (2.7%)	82 (4.2%)	76 (3.6%)	45 (2.3%)	42 (2.2%)	44 (2.4%)	32 (1.7%)	21 (1.1%)	45 (2.9%)	56 (3.0%)	11.1%
	Men	114 (5.5%)	165 (8.4%)	159 (7.5%)	117 (5.9%)	101 (5.2%)	107 (5.8%)	75 (3.9%)	48 (2.6%)	67 (4.3%)	64 (3.4%)	−38.2%
	PRs (95% CI)	1.24 (0.99, 1.57)	1.11 (0.92, 1.35)	1.08 (0.89, 1.32)	0.93 (0.72, 1.21)	0.93 (0.72, 1.21)	0.91 (0.70, 1.17)	0.89 (0.66, 1.20)	0.88 (0.61, 1.26)	0.90 (0.71, 1.14)	1.16 (0.95, 1.42)	
Professor^c^	Women	18 (0.9%)	14 (0.7%)	19 (0.9%)	23 (1.2%)	25 (1.3%)	23 (1.2%)	23 (1.2%)	25 (1.4%)	0 (0.0%)	0 (0.0%)	−100.0%
	Men	105 (5.0%)	115 (5.9%)	111 (5.3%)	118 (6.0%)	118 (6.1%)	101 (5.5%)	97 (5.0%)	102 (5.6%)	0 (0.0%)	0 (0.0%)	−100.0%
	PRs (95% CI)	0.52 (0.34, 0.80)	0.34 (0.21, 0.57)	0.47 (0.31, 0.71)	0.53 (0.36, 0.78)	0.54 (0.38, 0.77)	0.56 (0.39, 0.82)	0.56 (0.38, 0.81)	0.55 (0.39, 0.79)	n/a	n/a	
Total Leadership Position	Women	571 (17.3%)	592 (16.6%)	638 (17.7%)	583 (16.3%)	610 (16.2%)	590 (16.4%)	647 (17.0%)	630 (16.4%)	679 (20.7%)	753 (20.6%)	19.1%
	Men	1427 (21.2%)	1464 (20.4%)	1474 (20.7%)	1385 (19.8%)	1338 (19.3%)	1257 (19.7%)	1292 (19.8%)	1199 (18.8%)	869 (18.8%)	933 (19.2%)	−9.4%
	PRs (95% CI)	0.84 (0.78, 0.91)	0.84 (0.78, 0.91)	0.88 (0.82, 0.94)	0.80 (0.74, 0.87)	0.87 (0.81, 0.93)	0.86 (0.80, 0.93)	0.89 (0.83, 0.95)	0.90 (0.84, 0.96)	1.07 (1.00, 0.14)	1.05 (0.99, 1.12)	

*Note:* Data are displaying as *N* (count) and percentage (%). The percentage is calculated on the total of women + men for the corresponding years. 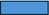: PRs < 0.50; 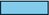: 0.50 ≤ PRs < 0.80; 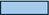: 0.80 ≤ PRs < 0.99; 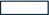: PRs = 1.00; 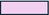: 1.0 < PRs < 1.20; 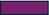: PRs ≥ 1.20.

Abbreviations: CI, confidence interval; PRs, prevalence ratios.

^a^ Percentage change is calculated between 2011–12 and 2022–23 percentages.

^b^ From 2021 to 2022 academic year, the category of ‘Dean’ was further subdivided into ‘Dean’, ‘Dean, Acting Interim’ and ‘Dean Emeritus’. For the purpose of this analysis, all three leadership positions were combined under the single category of ‘Dean’.

^c^ The ADEA U.S. Dental School Faculty Census included the category of ‘Professor’ to denote Professor Emeritus/Full Professor serving in administrative capacities.

**FIGURE 1:**
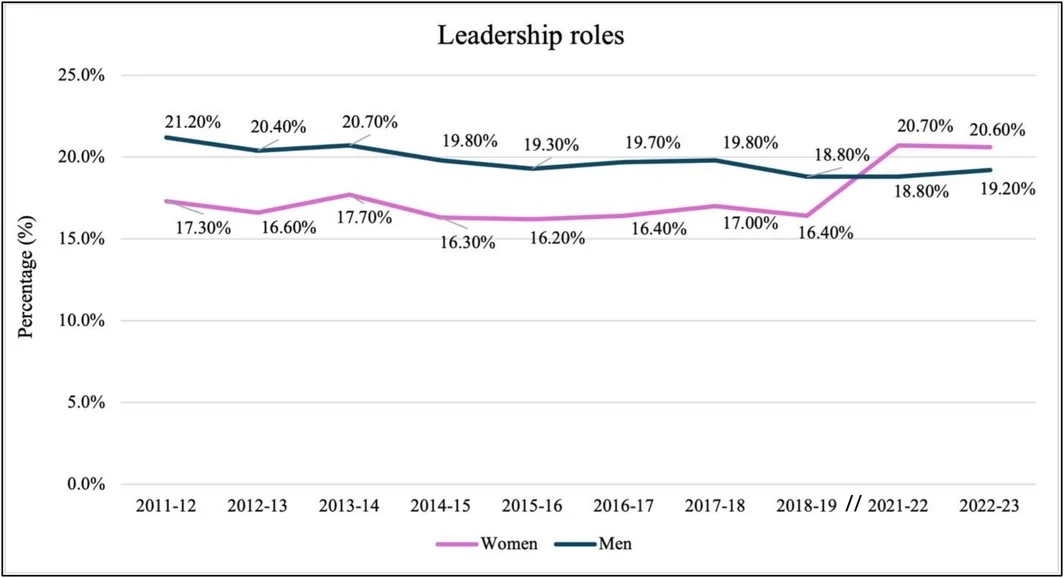
Proportion of faculty holding leadership roles over time.

Among the leadership positions, women were consistently overrepresented compared to men as allied dental program directors (PRs from 2.71 to 3.27), other directors (PRs from 1.10 to 2.05). Nevertheless, they were underrepresented across most academic years among division directors (PRs from 0.40 to 0.72, excepts for the years from 2014–15 to 2016–17), department chairs (PRs from 0.57 to 0.81, except for the 2022–23 academic year) and professors with administrative capacities (PRs from 0.34 to 0.56). Women were consistently underrepresented also in dean positions during the academic years 2012–13, 2017–18, 2021–22 and 2022–23 (PRs from 0.46 to 0.62); conversely, their underrepresentation in the remaining years was not statistically significant, as the 95% CI included 1. For roles such as assistant dean, associate dean, clinic director, program director and other administrative positions, the PRs fluctuated over time, with some years showing PRs above 1 and other below. However, in mostly all cases, the 95% CI included 1, indicating no statistically significant gender differences in representation during the study period.

### 3.2 Leadership Roles According to Academic Rank

Over the 12‐year period, notable gender‐based differences were observed in leadership roles across academic ranks. The proportion of women holding leadership position while in junior academic ranks consistently increased across nearly all roles, with a 68.8% increase in total representation compared to only 2.8% increase for men (Figure 2). Women assistant professors with leadership roles showed the most sustained gender gap, with PRs ranging from 1.23 (95% CI 1.04, 1.38) to 1.56 (95% CI 1.35, 1.80), indicating that women were significantly overrepresented relative to men through the period (Table 2). In contrast, women holding leadership positions remained significantly underrepresented at the senior ranks across all years (PRs ranging from 0.63 to 0.80, Figure 2), although the proportion of women increased by 50.0% by 2022–23, while men decreased by nearly 29.0%. Specifically, among associate professors, women's representation increased by 49.0% while men's decreased by 12.3% (PRs between 1.07 and 1.30). Nevertheless, women were consistently underrepresented among professors (PRs between 0.56 and 0.74) and professor emeritus (PRs between 0.44 and 0.55, except for the last 2 years which were not statistically meaningful due to very small numbers, Table 2).

**FIGURE 2:**
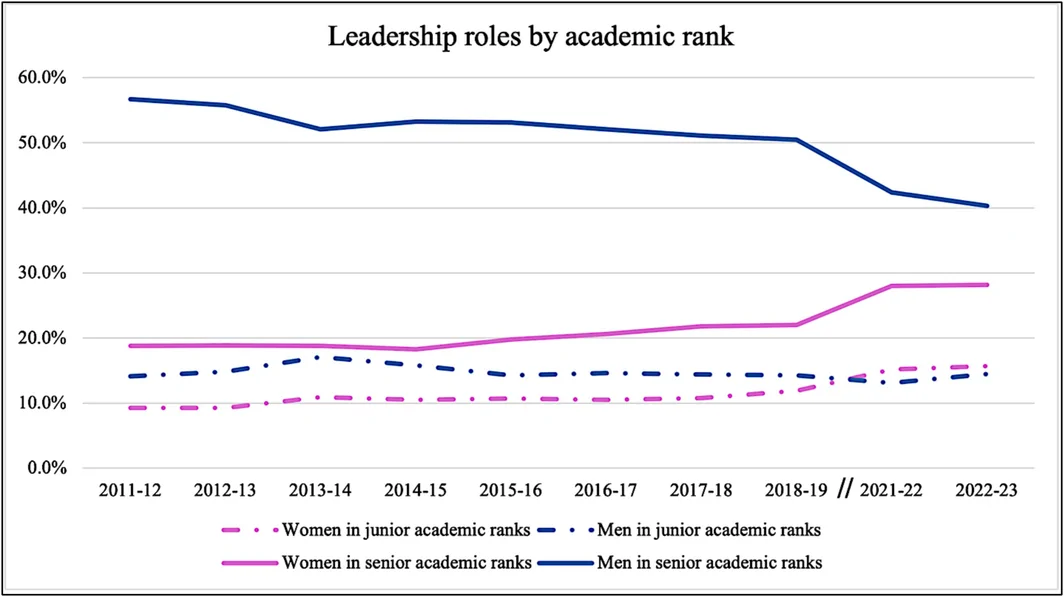
Leadership roles according to academic rank distribution between women and men. Those who answered either ‘No response’ (range from 0.0% to 0.2%), ‘Other’ (range from 0.3% to 0.8%), or ‘Teaching/Research Associate or Assistant Professor’ (range from 0.0% to 0.9%) regarding their academic rank were excluded from the current analysis. Junior academic rank: Assistant professor, instructor, lecturer; Senior academic rank: Associate professor, professor, professor emeritus.

**TABLE 2:** Proportion of faculty holding leadership roles according to gender distribution and academic rank over time.

Academic rank	Gender	2011–12	2012–13	2013–14	2014–15	2015–16	2016–17	2017–18	2018–19	2021–22	2022–23	% change^a^
Junior academic rank												
Instructor	Women	15 (0.8%)	16 (0.8%)	28 (1.3%)	19 (1.0%)	23 (1.2%)	22 (1.2%)	23 (1.2%)	19 (1.0%)	14 (0.9%)	21 (1.2%)	50.0%
	Men	21 (1.1%)	27 (1.3%)	56 (2.7%)	31 (1.6%)	24 (1.2%)	23 (1.2%)	22 (1.1%)	22 (1.2%)	9 (0.6%)	15 (0.9%)	−18.2%
	PRs (95% CI)	1.47 (0.99, 2.18)	1.30 (0.88, 1.93)	1.11 (0.81, 1.51)	1.29 (0.90, 1.85)	1.59 (1.17, 2.14)	1.55 (1.14, 2.11)	1.55 (1.16, 2.08)	1.36 (0.97, 1.90)	1.40 (1.00, 1.95)	1.32 (1.00, 1.75)	
Lecturer	Women	1 (0.1%)	4 (0.2%)	5 (0.2%)	5 (0.3%)	5 (0.3%)	5 (0.3%)	4 (0.2%)	4 (0.2%)	0 (0.0%)	1 (0.1%)	0%
	Men	4 (0.2%)	7 (0.3%)	6 (0.3%)	5 (0.3%)	6 (0.3%)	4 (0.2%)	3 (0.2%)	3 (0.2%)	0 (0.0%)	0 (0.0%)	−100.0%
	PRs (95% CI)	0.70 (0.12, 4.04)	1.27 (0.58, 2.77)	1.51 (0.79, 2.89)	1.69 (0.91, 3.16)	1.46 (0.76, 2.79)	1.75 (0.97, 3.14)	1.72 (0.90, 3.27)	1.66 (0.87, 3.17)	n/a	n/a	
Assistant Professor	Women	170 (8.5%)	171 (8.3%)	198 (9.4%)	183 (9.3%)	180 (9.2%)	167 (9.0%)	183 (9.4%)	194 (10.6%)	222 (14.3%)	244 (14.4%)	64.4%
	Men	257 (12.9%)	270 (13.1%)	300 (14.2%)	275 (14.0%)	248 (12.7%)	243 (13.2%)	255 (13.2%)	237 (13.0%)	194 (12.5%)	231 (13.6%)	5.4%
	PRs (95% CI)	1.56 (1.35, 1.80)	1.49 (1.29, 1.72)	1.46 (1.28, 1.67)	1.51 (1.31, 1.74)	1.49 (1.30, 1.71)	1.38 (1.20, 1.59)	1.35 (1.18, 1.55)	1.44 (1.27, 1.64)	1.32 (1.18, 1.48)	1.23 (1.04, 1.38)	
Total junior academic rank	Women	186 (9.3%)	191 (9.3%)	231 (10.9%)	207 (10.5%)	208 (10.7%)	194 (10.5%)	210 (10.8%)	217 (11.9%)	236 (15.2%)	266 (15.7%)	68.8%
	Men	282 (14.1%)	304 (14.8%)	362 (17.1%)	311 (15.8%)	278 (14.3%)	270 (14.6%)	280 (14.4%)	262 (14.3%)	203 (13.1%)	246 (14.5%)	2.8%
	PRs (95% CI)	1.58 (1.37, 1.82)	1.50 (1.31, 1.73)	1.45 (1.28, 1.66)	1.54 (1.34, 1.77)	1.56 (1.36, 1.78)	1.46 (1.28, 1.67)	1.42 (1.25, 1.62)	1.48 (1.30, 1.68)	1.35 (1.20, 1.51)	1.26 (1.13, 1.41)	
Senior academic rank												
Associate Professor	Women	208 (10.4%)	205 (10.0%)	203 (9.6%)	175 (8.9%)	184 (9.4%)	191 (10.3%)	218 (1.2%)	216 (11.8%)	241 (15.6%)	263 (15.5%)	49.0%
	Men	405 (20.3%)	390 (19.0%)	383 (18.1%)	354 (18.0%)	346 (17.8%)	325 (17.6%)	345 (17.8%)	323 (17.7%)	276 (17.8%)	302 (17.8%)	−12.3%
	PRs (95% CI)	1.30 (1.12, 1.49)	1.30 (1.31, 1.50)	1.22 (1.06, 1.39)	1.17 (1.01, 1.35)	1.16 (1.00, 1.33)	1.24 (1.07, 1.42)	1.24 (1.09, 1.42)	1.25 (1.10, 1.42)	1.10 (0.98, 1.23)	1.07 (0.96, 1.20)	
Professor	Women	147 (7.4%)	162 (7.9%)	171 (8.1%)	161 (8.2%)	173 (8.9%)	164 (8.9%)	179 (9.2%)	159 (8.7%)	190 (12.3%)	214 (12.6%)	70.3%
	Men	613 (30.7%)	621 (30.2%)	586 (27.7%)	562 (28.6%)	555 (28.5%)	521 (28.2%)	534 (27.5%)	489 (26.7%)	378 (24.4%)	378 (22.3%)	−27.4%
	PRs (95% CI)	0.56 (0.48, 0.67)	0.61 (0.52, 0.72)	0.66 (0.56, 0.76)	0.66 (0.56, 0.77)	0.66 (0.57, 0.77)	0.64 (0.55, 0.74)	0.66 (0.57, 0.76)	0.62 (0.53, 0.72)	0.67 (0.59, 0.76)	0.74 (0.65, 0.84)	
Professor Emeritus	Women	21 (1.1%)	21 (1.0%)	23 (1.1%)	25 (1.3%)	29 (1.5%)	26 (1.4%)	25 (1.3%)	27 (1.5%)	2 (0.1%)	1 (0.1%)	−90.9%
	Men	114 (5.7%)	136 (6.6%)	132 (6.3%)	133 (6.8%)	134 (6.9%)	116 (6.3%)	111 (5.7%)	112 (6.1%)	2 (0.1%)	3 (0.2%)	−96.5%
	PRs (95% CI)	0.53 (0.35, 0.79)	0.44 (0.30, 0.67)	0.47 (0.32, 0.69)	0.51 (0.36, 0.74)	0.55 (0.39, 0.77)	0.55 (0.39, 0.79)	0.53 (0.37, 0.76)	0.54 (0.39, 0.77)	1.14 (0.43, 3.04)	0.56 (0.10, 3.07)	
Total senior academic rank	Women	376 (18.8%)	388 (18.9%)	397 (18.8%)	361 (18.3%)	386 (19.8%)	381 (20.6%)	422 (21.8%)	402 (22.0%)	433 (28.0%)	478 (28.2%)	50.0%
	Men	1132 (56.7%)	1147 (55.8%)	1101 (52.1%)	1049 (53.3%)	1035 (53.1%)	962 (52.1%)	990 (51.1%)	924 (50.5%)	656 (42.4%)	683 (40.3%)	−28.9%
	PRs (95% CI)	0.63 (0.54, 0.72)	0.65 (0.56, 0.74)	0.68 (0.59, 0.77)	0.64 (0.56, 0.74)	0.64 (0.56, 0.73)	0.68 (0.60, 0.78)	0.70 (0.61, 0.79)	0.67 (0.59, 0.76)	0.74 (0.66, 0.83)	0.80 (0.72, 0.89)	
Other												
Teaching/Research Associate or Assistant	Women	2 (0.1%)	3 (0.1)	3 (0.1%)	9 (0.5%)	9 (0.5%)	8 (0.4%)	8 (0.4%)	5 (0.3%)	2 (0.1%)	0 (0.0%)	−100.0%
	Men	3 (0.2%)	2 (0.1%)	3 (0.1%)	15 (0.8%)	17 (0.9%)	17 (0.9%)	12 (0.6%)	5 (0.3%)	0 (0.0%)	0 (0.0%)	−100.0%
	PRs (95% CI)	1.40 (0.48, 4.12)	2.09 (1.02, 4.29)	1.66 (0.74, 3.70)	1.27 (0.75, 2.14)	1.11 (0.65, 1.89)	1.00 (0.56, 1.78)	1.20 (0.70, 2.06)	1.46 (0.78, 2.71)	n/a	n/a	
Other	Women	6 (0.3%)	10 (0.5%)	7 (0.3%)	6 (0.3%)	7 (0.4%)	7 (0.4%)	7 (0.4%)	6 (0.3%)	6 (0.4%)	5 (0.3%)	0.0%
	Men	8 (0.4%)	11 (0.5%)	8 (0.4%)	10 (0.5%)	8 (0.4%)	8 (0.4%)	10 (0.5%)	8 (0.4%)	10 (0.6%)	14 (0.8%)	100.0%
	PRs (95% CI)	1.51 (0.82, 2.77)	1.67 (1.06, 2.62)	1.66 (0.90, 2.67)	1.27 (0.67, 2.40)	1.50 (0.87, 2.58)	1.47 (0.85, 2.53)	1.24 (0.70, 2.19)	1.25 (0.68, 2.29)	0.85 (0.45, 1.61)	0.59 (0.28, 1.25)	
No response	Women	1 (0.1%)	0 (0.0%)	0 (0.0%)	0 (0.0%)	0 (0.0%)	0 (0.0%)	0 (0.0%)	2 (0.1%)	0 (0.0%)	4 (0.2%)	100.0%
	Men	2 (0.1%)	0 (0.0%)	0 (0.0%)	0 (0.0%)	0 (0.0%)	0 (0.0%)	0 (0.0%)	0 (0.0%)	0 (0.0%)	0 (0.0%)	−100.0%
	PRs (95% CI)	1.17 (0.24, 5.79)	n/a	n/a	n/a	n/a	n/a	n/a	n/a	n/a	n/a	

*Note:* Data are displaying as *N* (count) and percentage (%). The percentage is calculated on the total of women + men for the corresponding years. 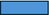: PRs < 0.50; 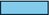: 0.50 ≤ PRs < 0.80; 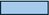: 0.80 ≤ PRs < 0.99; 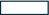: PRs = 1.00; 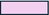: 2.0 < PRs < 1.20; 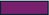: PRs ≥ 1.20.

Abbreviations: CI, confidence interval; PRs, prevalence ratios.

^a^ Percentage change is calculated between 2011–12 and 2022–23 percentages.

### 3.3 Leadership Roles According to Age Group

Among respondents holding leadership roles who self‐reported their age (on average 1853.5 per year, from 1901 in 2011–12 to 1547 in 2022–23), a sub‐analysis was conducted by age group (Table 3). Women between the ages of 20 and 59 years old holding leadership positions were overrepresented compared to men (PRs between 1.32 and 1.86). Conversely, women above the age of 60 holding leadership positions were severely or moderately underrepresented compared to their male counterparts (PRs between 0.47 and 0.58).

**TABLE 3:** Leadership roles by age group between women and men.

Age group	Gender	2011–12	2012–13	2013–14	2014–15	2015–16	2016–17	2017–18	2018–19	2021–22	2022–23
Age 20–39	Women	38 (2.0%)	42 (2.1%)	47 (2.2%)	53 (2.7%)	56 (2.9%)	50 (2.7%)	60 (3.1%)	63 (3.5%)	77 (5.2%)	78 (5.0%)
	Men	40 (2.1%)	52 (2.6%)	65 (3.1%)	61 (3.1%)	63 (3.2%)	53 (2.9%)	49 (2.6%)	50 (2.7%)	42 (2.8%)	56 (3.6%)
	PRs (95% CI)	1.76 (1.39, 2.24)	1.59 (1.26, 2.02)	1.42 (1.13, 1.78)	1.62 (1.32, 2.01)	1.56 (1.27, 1.91)	1.58 (1.28, 1.95)	1.72 (1.43, 2.06)	1.70 (1.42, 2.03)	1.53 (1.32, 1.78)	1.32 (1.75, 2.17)
Age 40–59	Women	333 (17.5%)	334 (16.6%)	367 (17.4%)	305 (15.5%)	295 (15.2%)	285 (15.5%)	312 (16.4%)	311 (17.1%)	390 (26.3%)	427 (27.6%)
	Men	544 (28.6%)	519 (25.7%)	511 (24.3%)	484 (24.6%)	451 (23.2%)	410 (22.3%)	442 (23.2%)	413 (22.7%)	354 (23.9%)	364 (23.5%)
	PRs (95% CI)	1.86 (1.61, 2.16)	1.85 (1.61, 2.12)	1.90 (1.66, 2.16)	1.64 (1.43, 1.88)	1.52 (1.34, 1.73)	1.56 (1.37, 1.78)	1.48 (1.30, 1.67)	1.51 (1.33, 1.71)	1.47 (1.30, 1.65)	1.50 (1.33, 1.68)
Age > 60	Women	171 (9.0%)	205 (10.2%)	224 (10.6%)	225 (13.1%)	255 (13.1%)	251 (13.6%)	263 (13.8%)	250 (13.7%)	187 (12.6%)	195 (12.6%)
	Men	775 (40.8%)	865 (42.9%)	893 (42.4%)	840 (42.7%)	823 (42.4%)	793 (43.1%)	779 (40.9%)	735 (40.3%)	433 (29.2%)	427 (27.6%)
	PRs (95% CI)	0.47 (0.40, 0.54)	0.48 (0.42, 0.56)	0.48 (0.42, 0.55)	0.53 (0.46, 0.61)	0.58 (0.51, 0.67)	0.57 (0.50, 0.66)	0.59 (0.51, 0.67)	0.57 (0.50, 0.65)	0.56 (0.49, 0.64)	0.57 (0.50, 0.65)

*Note:* Data are displaying as count (*N*) and percentage (%). The percentage is calculated on the total of women + men for the corresponding years. 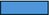: PRs < 0.50; 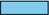: 0.50 ≤ PRs < 0.80; 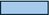: 0.80 ≤ PRs < 0.99; 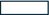: PRs = 1.00; 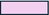: 3.0 < PRs < 1.20; 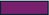: PRs ≥ 1.20.

Abbreviations: CI, confidence interval; PRs, prevalence ratios.

## 4 Discussion

The authors of this study observed a gradual but steady rise in the proportion of women relative to men undertaking leadership roles in dental education, across the study period of 2011–2023. Women assistant deans rose from 1.1% in 2011–2012 to 3.1% in 2022–2023. Women associate deans similarly rose from 3.3% to 6.4%. However, the proportion of women deans only marginally increased from 0.6% to 0.9%. This slow climb is consistent with findings in other areas of academic healthcare. In the past decade within academic medicine, there has been a similarly marginal rise in the percentage of US medical school deans that are women [23].

The only two leadership roles in our results where women consistently appeared overrepresented were as allied program directors and ‘other directors’. This may reflect how allied dental programs, like nursing programs in medicine [24], tend to be driven toward women more than men. Unlike the more balanced gender demographics among dental students [1], women make up over 90% of enrollees in dental assisting, dental hygiene and dental laboratory technology programs [25].

On a broader note, the trends observed also suggest that women, when they do hold leadership roles, disproportionately hold roles that contribute to the educational function of the school, and not roles that contribute to corporate strategic direction, policy enforcement and representation of the school to stakeholders and university leadership. This is another common observation in academic medicine. The proportion of women that hold positions like assistant dean, tend to be higher positions like associate dean, vice dean and dean [26].

Interestingly, when we observed academic rank as a variable, women with junior ranks were more commonly undertaking leadership roles. Across the study period, women with assistant professor ranks consistently overrepresented men in leadership roles. Contrarily, women with senior ranks held leadership roles less frequently than men. This may be part of a broader, cultural and generational shift toward more equitable workplaces. Junior rank faculty are usually faculty who have recently entered the academic workforce. One possibility is that resources for career advancement may be more accessible to faculty just beginning their careers. This shift might even extend to mindset. Demographic research and polling show that people aged 30 and under are more likely to value workplace equity and show a statistically significant preference for joining a workplace that invests in diversity, equity and inclusion [27, 28]. This could explain why our results demonstrate women aged 20–39, and 40–59 are more frequently holding leadership positions than women aged 60 and over. Similarly, our prior work [3] demonstrated that in recent years, women aged 20–39 have started to overrepresent men in senior academic rank positions. On the other hand, the severe underrepresentation of women aged 60 and over with leadership roles may reflect a low retention of women in senior academic ranks [3]. There is a persistent lack of effort to retain women faculty in dental education, especially so with what Garcia et al. [15] describe as the ‘motherhood penalty’. Women express their professional productivity tends to be negatively impacted by outdated institutional policies on parental leave, resulting in a struggle to balance caregiving commitments with work.

In 2024, a study surveying women dental faculty about career advancement and their experiences of barriers in the way of that advancement [29]. Here too, a lack of work‐life balance was a common theme. Women are often tasked with additional work responsibilities without accommodations to level the imbalance. Answers from survey participants underscored the need for change to restrictive institutional policies around parental leave and remote work flexibility. The same authors did a similar survey [30] almost a decade earlier and when compared to their more recent results, more women hold leadership positions in 2023 (76%) than 2015 (53%), mirroring the upward trend noted in our study. In this study, over 60% of women who were in administrative leadership roles never received extra remuneration with their leadership roles. From the lived experiences of these women, the authors noted recurring themes: the stigma of being a woman leader in ‘an old boys' club’, a systemically patriarchal leadership structure, racial bias around women of colour in positions of power and consistently lower salaries and scarce appreciation compared to their male counterparts.

Gender equity in the workplace is to the ultimate benefit of all parties involved. Women and men tend to have inherently different leadership styles [31, 32]. Diversifying academic leadership will encourage and support leadership styles that value and support collaborative and equitable work environments, unlike the patriarchal, hierarchical structures that pervade institutional and academic healthcare. Diverse, collaborative groups are more likely to be productive [33], but this change presently appears scarce in our profession.

Some of our findings suggest a promising upward trend in gender equity and highlight the work leadership programs, DEI initiatives, mentorship resources and peer networking opportunities do to pave the way for more women to take on leadership positions in academic healthcare [12, 34, 35, 36]. To return to the idea described early in this paper, it is only when women see other women in positions of leadership, influence and change that these positions even feel within reach. Research supports this philosophy, showing that more women hold senior leadership positions in institutions led by women as deans, in both academic medicine [26] and dentistry [37]. Our findings suggest that sustaining the efforts described above could help bridge the gender divide.

This study does have some limitations. Although adjustments were made for employment status, unmeasured factors such as years in academia and prior leadership training may have confounding associations.

Future research could include variables like race and ethnicity to comprehensively study equity in leadership. Many of the references in this paper were from research in academic medicine. Comparative analyses with other healthcare professions could determine whether the patterns we observe are field‐specific or reflect broader academic trends. Longitudinal studies of interventions like leadership development programs could identify the most effective strategies to foster diversity in leadership.

## 5 Conclusion

Within the limitations of this study, the authors observe a positive trend in the percentage of women holding leadership positions in academic dentistry over the study period of 12 years. Women appear to be consistently overrepresented as allied program directors and other, nonspecific directorships, but continue to be underrepresented across most academic years in many remaining leadership roles such as department chairs, division directors, and deans. Interestingly, with time, women faculty holding junior academic ranks and aged 20–59 years overrepresented men in leadership roles. While these are promising trends, there remain pervasive disparities in leadership positions with more influence and gravity. The need for equitable change in academic healthcare is real and immediate. Gender equity in academic healthcare requires that we transform the systems that shape it by reshaping policies and institutional norms to create leadership opportunities that are inclusive of all genders.

## Funding

The authors have nothing to report.

## Conflicts of Interest

The authors declare no conflicts of interest.

## Data Availability

The authors have nothing to report.
